# Paternal High-fat Diet Impairs Maternal Adaptations Essential for Normal Pregnancy

**DOI:** 10.1210/endocr/bqab143

**Published:** 2021-07-16

**Authors:** Hannah L Morgan, Adam J Watkins

**Affiliations:** Population and Lifespan Science, Faculty of Medicine, University of Nottingham, Nottingham NG7 2UH, UK

**Keywords:** Maternal adaptations, paternal diet, seminal plasma, uterine immune cells

The notion that our health as adults can be shaped by environmental factors acting on the way we develop in the womb is now well established. This area of research has become known as the Developmental Origins of Health and Disease (DOHaD) hypothesis. Initial observations from human cohort data defined a connection between disproportionate fetal growth and an increased likelihood of developing a range of cardiometabolic diseases in adult life. Since then, a detailed picture defining how even short-duration maternal insults can affect the cellular, genetic, and epigenetic information within the resultant offspring, shaping their future development and well-being has emerged. Out of this research has come the realization that specific “windows” during development and gestation display particular sensitivity to environmental perturbations. One such window is the “periconception” period, a time encompassing gamete maturation, fertilization of the oocyte, preimplantation embryo development, and implantation. Studies have shown that environmental perturbations, either in vivo or in vitro, during this sensitive time can predispose the resultant offspring to a range of cardiometabolic diseases (1).

To date, the majority of the research in the DOHaD field to date has focused on the connections between maternal lifestyle, environmental factors, and the health of her offspring. Over the past decade, a new focus within the DOHaD field has emerged, examining the paternal contribution to offspring health. Here, studies have shown that factors such as poor paternal diet, stress and advancing age can all impact significantly on the health and well-being of the offspring (2). Underlying these paternal programming effects are 2 main proposed mechanisms: the genomic and epigenetic information carried in the sperm and the impact of the seminal plasma on the maternal reproductive tract. While the impact of paternal health on sperm quality has been investigated in detail, the significance of the seminal plasma has been largely neglected. In their recent publication, Schjenken et al. use a mouse model to define how both paternal and maternal physiology respond to the impact of a paternal high-fat diet (3). Initially, Schjenken et al. observed that while the high-fat diet increased the levels of adiposity in males, it had minimal effect on sperm production or quality. However, analysis of the seminal vesicles showed a dramatic 65% increase in their volume in response to a high fat diet. Compositional analysis of the seminal plasma revealed decreased levels of several isoforms of TGFB and cytokines including chemokine (C-C motif) ligand 3 (CCL3), 5 (CCL5) and 11 (CCL11), chemokine (C-X-C motif) ligand 1 (CXCL1), interleukin 1 beta (IL1B), 6 (IL6), 10 (IL10), 17 (IL17) and tumor necrosis factor (TNF). All of these factors have been implicated in modulating the female reproductive tract immune response. As embryo implantation is regulated by a complex interplay of uterine remodeling, immune cell infiltration, and cross communication between the embryo and uterine tissue, changes in seminal plasma cytokine profiles could impact significantly on patterns of future development. Indeed, the group of Sarah Robertson has previously demonstrated the significance of seminal plasma for regulating on-going development in the mouse. Here, they used a model in which the seminal vesicles were surgically removed, they reported significant impairments in embryo development, uterine remodeling and long-term offspring health (4). Other studies have also shown that seminal plasma from males fed a suboptimal low-protein diet alone could program metabolic dysfunction in offspring, even when the sperm were from control diet–fed males (5). The current study pushed our understanding further by defining how the levels of specific factors in the seminal plasma change in response to diet. Data such as these could be used as a test to gauge the quality of the seminal plasma and the overall reproductive fitness of a male.

To establish how the maternal uterine environment responds to such changes in seminal plasma composition, the authors next profiled the expression of 632 inflammatory genes in females mated with control or high-fat diet–fed males. While mating alone upregulated the expression of multiple cytokines within the uterine tissue, exposure to a high-fat diet seminal plasma only altered the expression of just 2 genes, *Il23a* and *Nup85*. Gene ontology and analysis of upstream regulators revealed that pathways altered by TGFB1, IL10, and TNF, were the most differentially expressed within uterine samples exposed to the high-fat diet seminal plasma. Interestingly, the levels of all 3 of these factors were decreased in the seminal plasma of high-fat diet–fed males. These data suggest that the normal maternal inflammatory and immune cell responses that occur shortly after mating are dampened in response to paternal high-fat diet. A failure to initiate a sufficient maternal response after mating could be 1 factor linking paternal obesity with the reported decreases in male fertility (6).

To further elucidate the role of the seminal plasma on the maternal adaptations to pregnancy, Schjenken et al. profiled the populations of Treg cells within the uterine tissue after mating. Mating alone resulted in significant increases in the populations of CD4 + T cells and CD4 + FOXP3 + Treg cells, irrespective of the diet of the male. However, the number of these Treg cells was lower in females mated to high fat diet fed males, suggesting a blunting in their recruitment. Recruitment of T-reg cells into the uterine tissue in early pregnancy is critical as lower levels have been associated with implantation failure, miscarriage and gestational conditions like pre-eclampsia (7). The finding that the number of functionally competent Treg cells recruited to the uterus provides an essential mechanistic link between the poor paternal diet and a potential impairment in embryo implantation. Such changes have been linked to compromised ongoing fetal development and long-term offspring ill health (8).

Observations such as these reported by Schjenken et al. highlight the importance of understanding the impact of poor diet and lifestyle factors on male reproductive health. It is now becoming clear that the health of the father at the time of conception not only affects his chances of becoming a father, but it can affect the well-being of the mother as well as his unborn offspring also.

## Data Availability

Data sharing is not applicable to this article as no datasets were generated or analyzed during the current study.

## Abbreviations

DOHaD: Developmental Origins of Health and Disease

## Additional Information

**Disclosure Statement:** The authors have nothing to disclose.
